# Heritability of Clinically Diagnosed Obsessive-Compulsive Disorder Among Twins

**DOI:** 10.1001/jamapsychiatry.2024.0299

**Published:** 2024-04-03

**Authors:** David Mataix-Cols, Lorena Fernández de la Cruz, Jan C. Beucke, Elles De Schipper, Ralf Kuja-Halkola, Paul Lichtenstein, Josep Pol-Fuster

**Affiliations:** 1 Center for Psychiatry Research, Department of Clinical Neuroscience, Karolinska Institutet, and Stockholm Health Care Services, Region Stockholm, Stockholm, Sweden; 2 Institute for Systems Medicine, Department of Human Medicine, MSH Medical School Hamburg, Hamburg, Germany; 3 Department of Medical Epidemiology and Biostatistics, Karolinska Institutet, Stockholm, Sweden

## Abstract

This cohort study estimates the heritability of clinically diagnosed obsessive-compulsive disorder in a sample of twins.

Obsessive-compulsive disorder (OCD) is thought to be moderately heritable (around 40%-50%). Evidence comes from extended family pedigrees of individuals with clinically diagnosed OCD^1,2^ and, indirectly, from nonclinical twin studies of research volunteers self-reporting current obsessive-compulsive symptoms.^3^ These phenotypes overlap, but are not equivalent; it is possible that heritability estimates based on self-report data differ from estimates derived from clinician diagnoses. This is the first study, to our knowledge, to estimate the heritability of clinically diagnosed OCD from a sample of twins.

## Methods

The Swedish Ethical Review Authority approved the study without requiring informed consent because the included individuals were not identifiable at any time. This cohort study followed the STROBE reporting guideline.

We linked the Swedish Twin Registry,^4^ which includes 143 853 twins born between 1886 and 2008, with information on diagnoses from the National Patient Register (NPR), registered between 1973 and 2020. Data were analyzed from October 1, 2023, to November 1, 2023. Zygosity was determined using DNA testing or intrapair physical similarities. OCD cases were identified as the first recorded diagnosis (300.3 in *ICD-8*, 300D in *ICD-9*, or F42 in *ICD-10*) in the NPR after the age of 6 years to limit the risk of diagnostic misclassification. To allow for a minimum time for both members of a twin pair to receive an OCD diagnosis, the twins were only included if they were at least 12 years of age.

All twins contributed to the data analyses. We calculated tetrachoric correlations for monozygotic (MZ) and dizygotic (DZ) pairs and fitted structural equation models to estimate additive genetic, shared environmental, and unique environmental effects, assuming a liability-threshold model with OCD liability being normally distributed. We adjusted for sex and birth year (including linear and quadratic effects) in all models. Analyses were performed in OpenMx. A 1-sided *P* < .05 was significant.

## Results

We excluded 1543 twins who emigrated and 4900 who died before the age of 12 years or before 1979 and 5016 with unknown zygosity, resulting in a cohort of 132 394 twins (74 735 twin pairs: 57 659 complete and 17 076 incomplete). Median age at first diagnosis among all individuals with OCD was 22.34 (IQR, 16.19-34.57) years. Among MZ twins, there were 15 pairs (9 female) concordant and 199 pairs (119 female) discordant for OCD. Among DZ twins, there were 7 pairs (2 female; 3 opposite sex) concordant and 412 pairs (152 female; 168 opposite sex) discordant for OCD. Tetrachoric correlations for MZ and DZ twins were 0.52 (95% CI, 0.40-0.63) and 0.21 (95% CI, 0.07-0.34), respectively (Figure).

**Figure.**
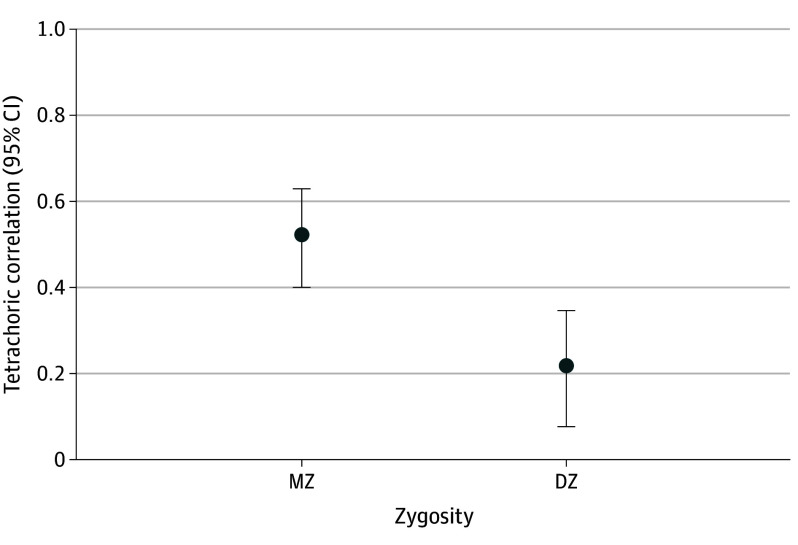
Tetrachoric Correlations for Clinically Diagnosed Obsessive-Compulsive Disorder in Monozygotic (MZ) and Dizygotic (DZ) Twins The MZ correlations are based on 22 493 twin pairs (18 767 complete pairs and 3726 incomplete pairs). The DZ correlations are based on 28 653 same-sex twin pairs (23 084 complete pairs and 5569 incomplete pairs) and 23 589 opposite-sex twin pairs (15 808 complete pairs and 7781 incomplete pairs).

Liability thresholds were equated across zygosity and twin order without loss in fit. Dropping the shared environment parameter did not result in a significant loss in fit. By contrast, dropping the additive genetic parameter resulted in a significantly worse fit. The Table shows parameter estimates and 95% CIs based on the best-fitting additive genetic–unique environmental model. Genetic factors accounted for 50% of the variance for OCD, with nonshared environmental factors (including measurement error) accounting for the other half.

**Table.** Model-Fitting Results and Parameter Estimates of Obsessive-Compulsive Disorder Liability Heritability, Adjusted for Sex and Birth Year^a^

Model	Model-fitting results						Parameter estimates, % (95% CI)		
	−2 Log-likelihood statistic	*df*	Δχ^2^ (Δ*df*)^b^	*P* value	Akaike information criterion	Model compared with	A	C	E
1. Saturated	8678.428	132 385	NA	NA	8696.428	NA	NA	NA	NA
2. ACE	8679.764	132 388	1.336 (3)	.72	8693.764	1	59.9 (24.4 to 95.8)	−8.6 (−39.2 to 20.0)	48.7 (38.1 to 60.8)
3. AE^c^	8680.094	132 389	0.330 (1)	.56	8692.094	2	50 (38.8 to 60.1)	NA	50 (40.0 to 61.3)
4. CE	8690.732	132 389	10.968 (1)	<.001	8702.732	2	NA	35.9 (26.9 to 44.3)	64.1 (55.7 to 73.1)
5. E	8743.899	132 390	64.135 (2)	<.001	8753.899	2	NA	NA	100 (100 to 100)

Abbreviations: A, additive genetic; C, shared environmental; E, nonshared environmental; NA, not applicable.

^a^ Variance components are not constrained to be positive in the presented models.

^b^ The difference in −2 log-likelihood statistic and *df* between the submodel and the full model.

^c^ AE was the best-fitting model.

## Discussion

The heritability of clinically diagnosed OCD in this study was similar to that reported in previous nonclinical twin studies of individuals self-reporting symptoms^3^ despite only partially overlapping constructs being measured. Environmental factors shared by twins growing up in the same family did not contribute to OCD liability, whereas nonshared environmental factors explained the remaining variance. The findings support the importance of identifying unique environmental factors implicated in OCD.^5^

Study strengths are the unique cohort of 633 twin pairs in which at least 1 twin had a formal diagnosis of OCD and the use of validated *ICD* codes.^6^ Limitations include insufficient power to examine sex differences in heritability and that the NPR does not capture individuals who do not seek help or who are only seen in primary care, which may affect the generalizability of the findings.

## Notes

Supplement. Data Sharing Statement
