# Supplementary material for: Heritability of Clinically Diagnosed Obsessive-Compulsive Disorder Among Twins
Source: JAMA Psychiatry. 2024 Apr 3;81(6):631–2. doi: 10.1001/jamapsychiatry.2024.0299 (PMC10993148; doi:10.1001/jamapsychiatry.2024.0299)
Supplement: Supplement. — Data Sharing Statement [file jamapsychiatry-e240299-s001.pdf]

Data Sharing Statement

Mataix-Cols. Heritability of Clinically Diagnosed Obsessive-Compulsive Disorder Among Twins. *JAMA Psychiatry*. Published April 03, 2024. doi:10.1001/jamapsychiatry.2024.0299

Data
Data available: No

Additional Information
Explanation for why data not available: Swedish register data cannot be shared.
